# PPARβ/δ Agonist GW0742 Modulates Microglial and Astroglial Gene Expression in a Rat Model of Temporal Lobe Epilepsy

**DOI:** 10.3390/ijms251810015

**Published:** 2024-09-17

**Authors:** Olga E. Zubareva, Adeliya R. Kharisova, Anna I. Roginskaya, Anna A. Kovalenko, Maria V. Zakharova, Alexander P. Schwarz, Denis S. Sinyak, Aleksey V. Zaitsev

**Affiliations:** Laboratory of Molecular Mechanisms of Neural Interactions, Sechenov Institute of Evolutionary Physiology and Biochemistry of RAS, 194223 Saint Petersburg, Russia; zubarevaoe@mail.ru (O.E.Z.); adeliaharisova.ah@gmail.com (A.R.K.); roganna5500@gmail.com (A.I.R.); kovalenko_0911@mail.ru (A.A.K.); zaharova-masha@yandex.ru (M.V.Z.); aleksandr.pavlovich.schwarz@gmail.com (A.P.S.); den67920405@yandex.ru (D.S.S.)

**Keywords:** gene expression, microglia, astroglia, neuroinflammation, temporal lobe epilepsy, PPAR β/δ agonists, GW0742, fitorine

## Abstract

The role of astroglial and microglial cells in the pathogenesis of epilepsy is currently under active investigation. It has been proposed that the activity of these cells may be regulated by the agonists of peroxisome proliferator-activated nuclear receptors (PPARs). This study investigated the effects of a seven-day treatment with the PPAR β/δ agonist GW0742 (Fitorine, 5 mg/kg/day) on the behavior and gene expression of the astroglial and microglial proteins involved in the regulation of epileptogenesis in the rat brain within a lithium–pilocarpine model of temporal lobe epilepsy (TLE). TLE resulted in decreased social and increased locomotor activity in the rats, increased expression of astro- and microglial activation marker genes (*Gfap, Aif1*), pro- and anti-inflammatory cytokine genes (*Tnfa*, *Il1b, Il1rn*), and altered expression of other microglial (*Nlrp3, Arg1*) and astroglial (*Lcn2, S100a10*) genes in the dorsal hippocampus and cerebral cortex. GW0742 attenuated, but did not completely block, some of these impairments. Specifically, the treatment affected *Gfap* gene expression in the dorsal hippocampus and *Aif1* gene expression in the cortex. The GW0742 injections attenuated the TLE-specific enhancement of *Nlrp3* and *Il1rn* gene expression in the cortex. These results suggest that GW0742 may affect the expression of some genes involved in the regulation of epileptogenesis.

## 1. Introduction

Epilepsy is a chronic and difficult-to-treat neurological disorder that affects millions of individuals worldwide [1]. This disease is defined by a predisposition to the occurrence of spontaneous epileptic seizures and may be accompanied by a range of psychiatric and neurological manifestations [2]. To date, more than 20 anti-epileptic drugs, for example, lamotrigine, carbamazepine, and phenytoin, have been introduced into clinical practice [3]. Despite the availability of a wide range of anticonvulsant drugs, up to 30% of patients with epilepsy remain refractory to treatment [4]. Accordingly, the identification of novel therapeutic agents is an urgent goal. In recent years, a considerable amount of research has been devoted to investigating the potential involvement of astro- and microglial cells in the pathogenesis of epilepsy. Neuroinflammation associated with increased astroglial and microglial cell activity plays an important role in the pathogenesis of epilepsy [5,6]. Pro-inflammatory proteins produced by glial cells, particularly interleukin-1β and tumor necrosis factor, have been shown to lower seizure thresholds in models of acute seizures [7,8] and to contribute to the development of chronic epileptic processes in the brain [9,10] and the formation of epilepsy-related behavioral disorders [11].

Reactive astro- and microgliosis represents one of the most distinctive histopathological alterations associated with epilepsy [12]. However, the mere activation of glial cells does not yet indicate an increase in the production of pro-inflammatory proteins. Astrocytes and microglia can exist in different functional states, or phenotypes, which are associated with the production of neurotoxic or neuroprotective proteins. In this regard, depending on the state, they may exert pro- or anti-epileptogenic effects. In previous years, the classification of astrocytes and microglia was a widely used method of distinguishing between the A1 and M1 phenotypes (pro-inflammatory) and the A2 and M2 phenotypes (protective) [13,14]. At present, this classification is considered too simplistic, and its use is currently being debated [14,15]. However, the view that, when examining the role of glial cells in various neuropathologies, it is essential to study the gene expression of both damaging and protective proteins remains unchanged [15].

Pharmacological agents that can inhibit pro-inflammatory processes while simultaneously activating the protective properties of glial cells represent a promising avenue of treatment for epilepsy [16]. In particular, peroxisome proliferator-activated receptor (PPAR) agonists exhibit these characteristics [14,17]. PPARs are nuclear receptor proteins that regulate the expression of genes essential for various metabolic processes, cell differentiation, and neuroinflammation [18]. There are three types of PPARs—α, β/δ, and γ. All of these receptor types are targets for free fatty acids and selected products of lipid metabolism, particularly eicosanoids [19]. Nevertheless, specific ligands have been identified for distinct subtypes of PPARs. For instance, PPARγ is activated by the prostaglandin PGJ2 [20], while PPARα is activated by leukotriene B4 [21]. Additionally, numerous fatty acids, particularly arachidonic acid derivatives, can bind to PPARβ/δ. However, the specific fatty acids that act as endogenous ligands for PPARβ/δ remain unresolved [22].

All types of PPARs are expressed in various cells throughout the body, including those in the brain. They are found in neurons, oligodendrocytes, astrocytes, as well as in relatively smaller amounts in the microglia [23,24,25]. Additionally, distinct patterns of distribution are observed for the three types of PPARs within different regions of the central nervous system (CNS) and among various cell types within the CNS [24].

The anti-inflammatory properties characteristic of the agonists of all types of PPARs are realized through the negative regulation of NF-κB, AP-1, and C/EBP-mediated signaling pathways [26]. Consequently, the effects of PPAR agonists are manifested by the suppression of oxidative stress, a reduction in the production of inducible NO synthase and pro-inflammatory cytokines, and an increase in the expression of anti-inflammatory genes [26]. The neuroprotective properties of synthetic PPAR agonists have been demonstrated in models of diverse neuropathologies, including epilepsy [27,28]. Meanwhile, in models of epilepsy and acute seizures, studies have primarily concentrated on examining the protective properties of PPARγ agonists [29,30,31,32]. There have been few studies conducted using PPARα agonists [33]. The effects of PPARβ/δ agonists have yet to be sufficiently studied.

The objective of this study was to examine the impact of the selective PPARβ/δ agonist GW0742 on the behavior and gene expression of the astro- and microglial proteins associated with epileptogenesis in the rat brain, using the lithium–pilocarpine model of temporal lobe epilepsy. GW0742, also known as GW610742 and Fitorine, is a selective PPARβ/δ agonist [34]. It is used in sports nutrition as a performance-enhancing and fat-burning drug. A number of studies have demonstrated the drug’s broad spectrum of action, including its ability to inhibit the interaction between the vitamin D receptor and steroid receptor coactivator 2 [35]. Additionally, GW0742 has been identified as a potential anti-diabetic drug [36]. GW0742 has been demonstrated to possess a marked anti-inflammatory effect, as evidenced by a number of research findings [37,38], including studies conducted in models of neuropathology [39]. The neuroprotective effects of GW0742 have also been demonstrated in models of neurodegenerative disease [40,41]. Nevertheless, the effects of GW0742 in models of epilepsy and seizures remain poorly understood.

## 2. Results

### 2.1. Survival and Weight Trend Analysis

**Figure 1:**
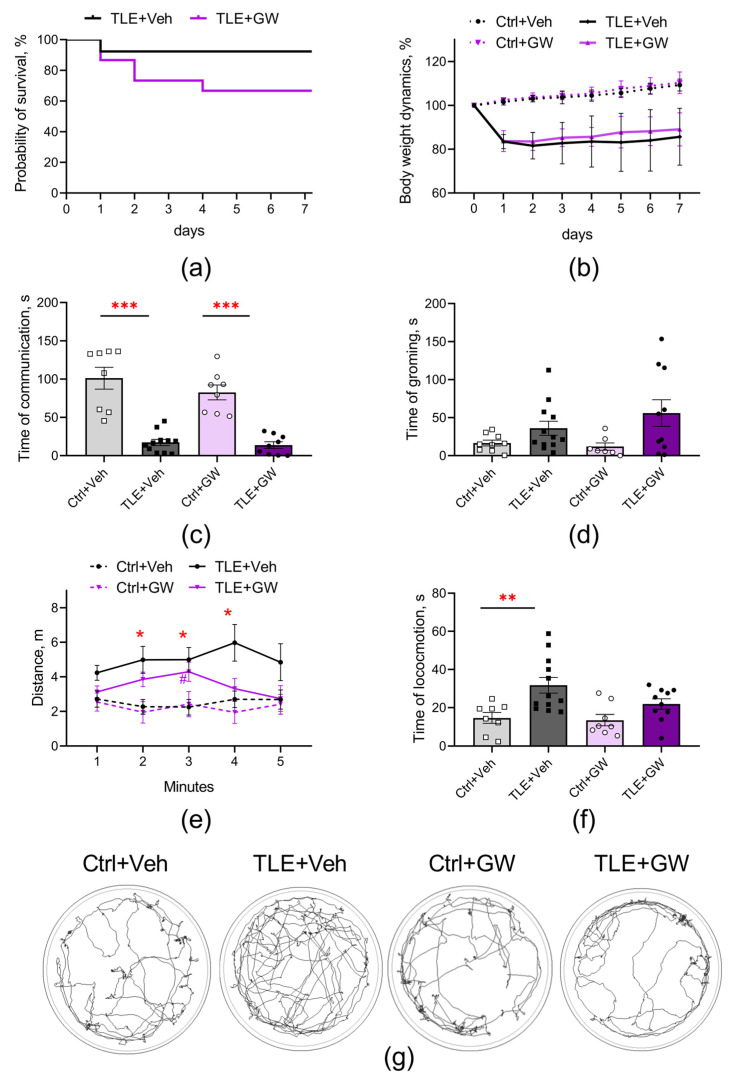
Effects of GW0742 treatment on survival and behavior of rats in a lithium-pilocarpine model of TLE. (**a**) Kaplan–Meier survival curves. (**b**) Body weight dynamics. Behavior of control and TLE rats in the social test (**c**) and open field test (**d**–**g**): (**c**) Communication time in the social test; (**d**) Grooming time; (**e**) Length of distance traveled; (**f**) Locomotion time; (**g**) Examples of tracks in the open field. Ctrl+Veh (*n* = 9), control rats without treatment; Ctrl+GW (*n* = 8), GW0742-treated control rats; TLE+Veh (*n* = 12), TLE rats; TLE+GW (*n* = 10), GW0742-treated TLE rats. Data are presented as mean and standard error of the mean. Each point represents the value of a single animal. Asterisks indicate significant differences between groups according to Sidak’s test: * *p* < 0.05; ** *p* < 0.01; *** *p* < 0.001.

The studies were conducted using a lithium–pilocarpine model of TLE on male Wistar rats aged 7–8 weeks. GW0742 (5 mg/kg) was administered once daily for a week, following the induction of status epilepticus with pilocarpine. The mortality rates observed in the initial period following the induction of status epilepticus with pilocarpine were 8% in the control group of rats and 33% in the GW0742-treated group. Nevertheless, a comparison of the survival curves (log-rank test, Figure 1a) reveals no statistically significant differences between the groups (χ2 = 2.47; *p* = 0.16). These results are consistent with those previously reported for this model. Mortality in this model is typically 20–30% of rats [9].

The initial weight of the animals did not differ significantly between the groups. Following the administration of pilocarpine, a decrease was observed, with a reduction of 12–24% (Figure 1b, two-way ANOVA, TLE factor: F_(1, 35)_ = 118; *p* < 0.001;). GW0742 had no effect on the weight dynamics of the control and TLE rats (treatment factor: F_(1, 35)_ = 0.97; *p* = 0.33).

### 2.2. Behavior

The results of the social interaction test (Figure 1c) revealed a significant reduction in communicative activity in the TLE rats, with a 6-fold decrease observed (two-way ANOVA, TLE factor—F_(1, 32)_ = 84; *p* < 0.001). Furthermore, the TLE rats exhibited increased psycho-emotional tension in the open field test, evidenced by an elevated duration of grooming behavior (Figure 1d; F_(1, 34)_ = 7.7; *p* < 0.01). GW0742 had no effect on these changes.

Hyperactivity represents one of the most salient behavioral disturbances that is consistently observed in the lithium–pilocarpine model [9,42]. Indeed, the TLE rats exhibited increased locomotor activity (Figure 1), as evidenced by the elevated length of distance traveled in the open field test (Figure 1e, mixed ANOVA, TLE factor: F_(1, 35)_ = 5.01; *p* < 0.01) and by the increased locomotion time (Figure 1f, two-way ANOVA, TLE factor: F_(1, 35)_ = 11.8; *p* < 0.01). A post hoc analysis employing Sidak’s test demonstrated that the statistically significant differences were between the untreated control and TLE groups (*p* < 0.05) in distance and locomotion time. Animals injected with GW showed no statistically significant differences from the corresponding control. The results showed that GW0742 attenuates some pathological processes occurring during the development of TLE.

Accordingly, we proceeded to the second stage of the study, which entailed an examination of the impact of GW0742 on the gene expression of the astro- and microglial proteins that are implicated in epileptogenesis regulation.

### 2.3. Gene Expression Analysis of Glial Protein Genes at the mRNA and Protein Level

#### 2.3.1. Analysis of the Expression of Astrocyte and Microglia Activation Markers

**Figure 2:**
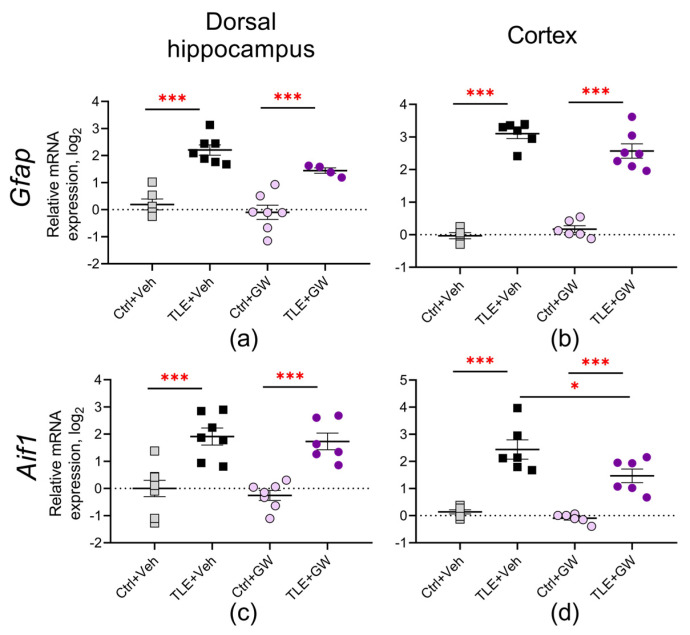
Relative gene expression of astrocyte activation marker *Gfap* (**a**,**b**) and microglial cell activation marker *Aif1* (**c**,**d**) in the dorsal hippocampus and temporal cortex of TLE and control rats. Ctrl+Veh (*n* = 7), control rats without treatment; Ctrl+GW (*n* = 7), GW0742-treated control rats; TLE+Veh (*n* = 6–7), TLE rats; TLE+GW *(n* = 6–7), GW0742-treated TLE rats. Data are presented as mean and standard error of the mean. Each point represents the value of a single animal. Asterisks indicate significant differences between groups according to Sidak’s test: * *p* < 0.05, *** *p* < 0.001.

The analysis of gene expression of the markers of astrocyte (*Gfap*) and microglia (*Aif1)* activation in the treated and untreated TLE rats (Figure 2) revealed a notable elevation in both markers in the dorsal hippocampus (TLE factor: *Gfap—*F_(1, 20)_ = 62, *p* < 0.001; *Aif1—*F_(1, 24)_ = 47, *p* < 0.001) and cortex (TLE factor: *Gfap*—F_(1, 20)_ = 280, *p* < 0.001; *Aif1—*F_(1, 20)_ = 75, *p* < 0.001) of the TLE rats. A significant effect of treatment by GW0742 was found using two-way ANOVA with respect to the *Gfap* gene expression in the dorsal hippocampus (treatment factor: F_(1, 20)_ = 5.4, *p* = 0.03) and *Aif1* gene expression in the cortex (treatment factor: F_(1, 20)_ = 7.3, *p* = 0.01). Post hoc analysis and ANOVA contrast method for *Aif1* also reveal significant differences between the TLE+Veh and TLE+GW groups (*p* < 0.05).

**Figure 3:**
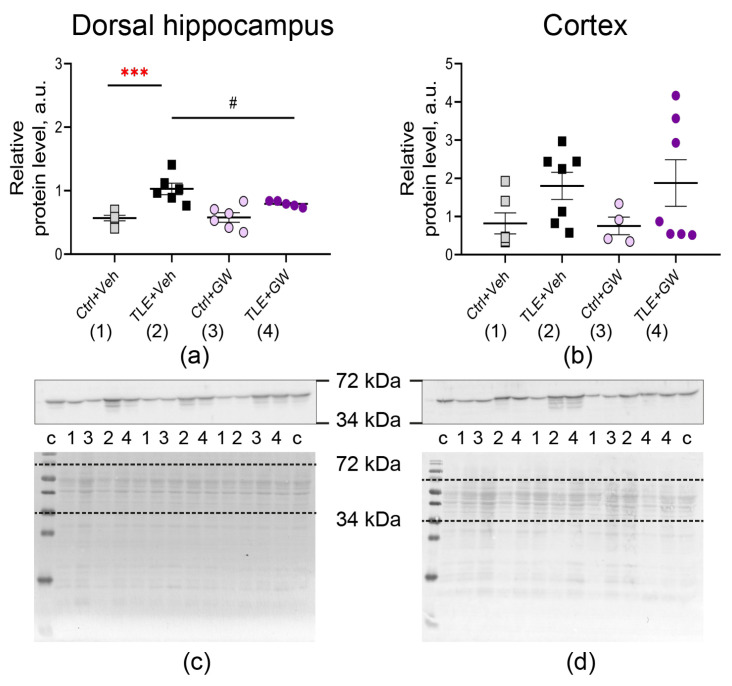
Changes in relative protein levels of the astrocyte marker GFAP in the dorsal hippocampus (**a**,**c**) and cortex (**b**,**d**) of rats. Ctrl+Veh (*n* = 6), control rats without treatment; Ctrl+GW (*n* = 4–6), GW0742-treated control rats; TLE+Veh (*n* = 6–7), TLE rats; TLE+GW (*n* = 6–7), GW0742-treated TLE rats. Data are presented as mean and standard error of the mean. Each point represents the value of a single animal. Asterisks indicate significant differences between groups according to Sidak’s test: ***—*p* < 0.001. ANOVA contrast method: # *p* < 0.05.

**Figure 4:**
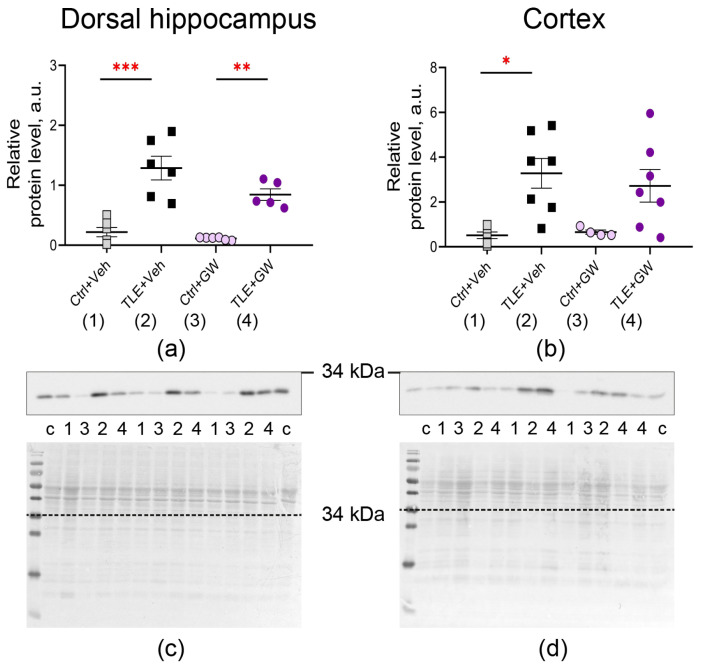
Changes in relative protein levels of the microglial cell marker IBA1 in the dorsal hippocampus (**a**,**c**) and cortex (**b**,**d**) of rats. Ctrl+Veh (*n* = 6), control rats without treatment; Ctrl+GW (*n* = 4–6), GW0742-treated control rats; TLE+Veh (*n* = 6–7), TLE rats; TLE+GW (*n* = 6–7), GW0742-treated TLE rats. Data are presented as mean and standard error of the mean. Each point represents the value of a single animal. Asterisks indicate significant differences between groups according to Sidak’s test: * *p* < 0.05, ** *p* < 0.01, *** *p* < 0.001.

The alterations in gene expression were confirmed at the protein level. We revealed an increase in GFAP (Figure 3, TLE factor: dorsal hippocampus—F_(1, 19)_ = 26; *p* < 0.0001; cortex—F_(1, 20)_ = 5.3; *p* = 0.03) and IBA1 production (Figure 4, TLE factor: dorsal hippocampus—F_(1, 19)_ = 57; *p* < 0.0001; cortex—F_(1, 20)_ = 16.1, *p* < 0.001).

Post hoc comparisons of GFAP protein levels (Figure 3) revealed significant differences between the untreated control group and the TLE group, as evidenced by Sidak’s test (*p* < 0.001). The ANOVA contrast method showed significant differences between the TLE+Veh and TLE+GW groups (*p* < 0.05). A significant effect of GW0742 treatment on IBA1 protein levels was found in the dorsal hippocampus (Figure 4a, two-way ANOVA, factor of treatment: F_(1, 19)_ = 5.3; *p* = 0.03). In the cortex of the untreated TLE animals, the IBA levels were significantly higher than in the controls (post hoc Sidak’s test, *p* = 0.01). However, no such differences were observed in the treated animal groups (*p* = 0.13).

These findings suggest that GW0742 attenuates, but does not completely block, the micro- and astroglial cell activation associated with epileptogenesis.

#### 2.3.2. Analysis of the Expression of Pro- or Anti-Inflammatory Cytokines

Given that the activation of astro- and microglial cells does not provide sufficient evidence to determine whether pro- or anti-inflammatory protein gene expression is predominant, we conducted further analysis of the gene expression of pro- and anti-inflammatory cytokines, which are characteristic of both astrocytes and microglia. Additionally, we examined the gene expression of pro-inflammatory and protective proteins that are specific to microglia or astrocytes.

**Figure 5:**
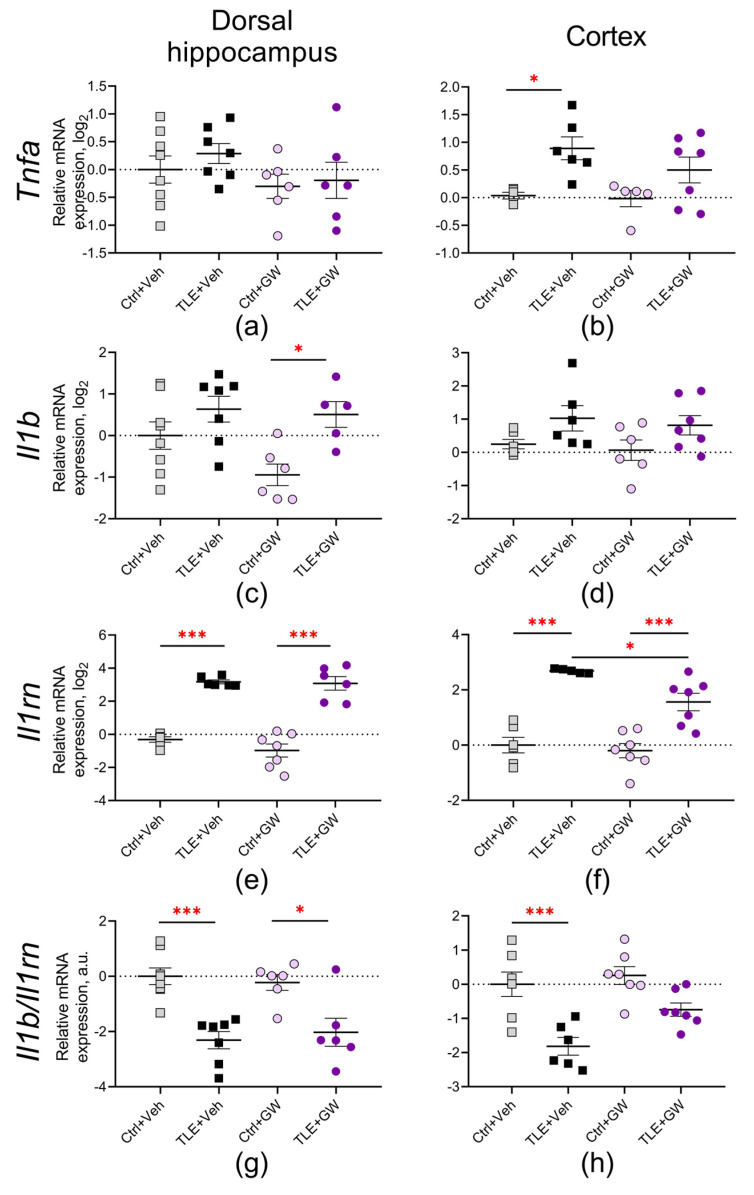
Relative gene expression of pro-inflammatory cytokines *Tnfa* and *Il1b* (**a**–**d**) and anti-inflammatory cytokine *Il1rn* (**e**,**f**) and their ratio (**g**,**h**) in the dorsal hippocampus and cortex of experimental and control rats. Ctrl+Veh (*n* = 7), control rats without treatment; Ctrl+GW (*n* = 6–7), GW0742-treated control rats; TLE+Veh (*n* = 6–7), TLE rats; TLE+GW (*n* = 5–7), GW0742-treated TLE rats. Data are presented as mean and standard error of the mean. Each point represents the value of a single animal. Asterisks indicate significant differences between groups according to Sidak’s test: * *p* < 0.05, *** *p* < 0.001.

The induction of TLE resulted in an increased expression of pro-inflammatory genes. *Tnfa* was upregulated in the cortex (TLE factor: F_(1, 19)_ = 12.4; *p* = 0.002) but not in the dorsal hippocampus (Figure 5a,b). There was a significant increase in the *Il1b* mRNA levels in both the dorsal hippocampus and cortex (F_(1, 22)_ = 10.7; *p* < 0.01 and F_(1, 21)_ = 6.73; *p* = 0.02; Figure 5c,d). The direction and severity of these changes were similar in the GW0742-treated and untreated rats. Two-way ANOVA revealed no significant effect of GW0742 administration on *Tnfa* and *Il1b* expression.

Additionally, there was a significant increase in the expression of the anti-inflammatory cytokine *Il1rn* (Figure 5e,f, TLE factor: dorsal hippocampus—F_(1, 22)_ = 151; *p* < 0.001; cortex—F_(1, 21)_ = 68; *p* < 0.001). Furthermore, the ratio of *Il1b* and *Il1rn* gene expression decreased (Figure 5g,h, dorsal hippocampus—F_(1, 23)_ = 34; *p* < 0.001; cortex—F_(1, 23)_ = 26; *p* < 0.001). This indicates that during the latent period of the lithium–pilocarpine model, compensatory mechanisms against the pathological processes remained highly active. Treatment with GW0742 resulted in a decrease in these changes in the cortex (factor of treatment for *Il1rn*: F_(1, 21)_ = 6.0; *p* = 0.02; for *Il1b/Il1rn*—F_(1, 23)_ = 5.8; *p* = 0.02). Post hoc Sidak’s test and ANOVA contrast method reveal significant differences between the TLE+Veh and TLE+GW groups *(p* < 0.05) for *Il1rn*. This may be attributed to the reduced severity of pathological changes observed in treated rats, which suggests that the activation of compensatory mechanisms was less pronounced.

#### 2.3.3. Analysis of the Expression of Microglial Pro-Inflammatory and Protective Proteins

**Figure 6:**
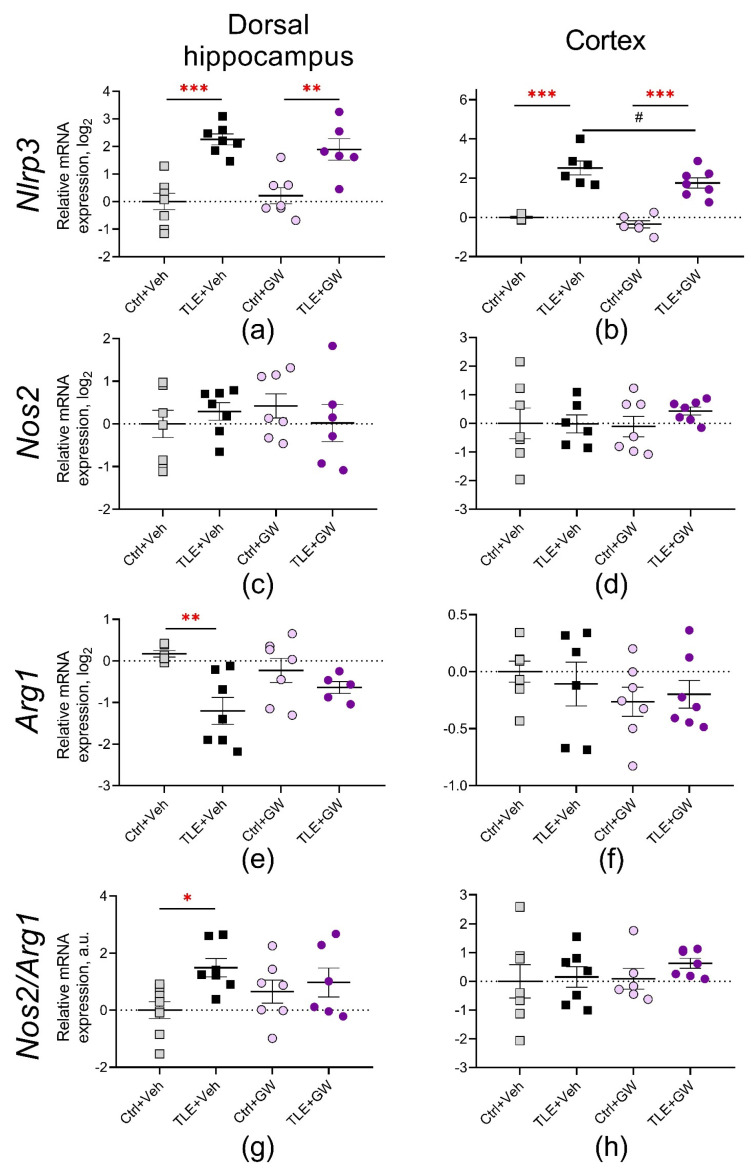
Relative gene expression of microglial pro-inflammatory (**a**–**d**) and protective (**e**,**f**) proteins and their ratio (**g**,**h**) in the dorsal hippocampus and cortex of experimental and control rats. Ctrl+Veh (*n* = 7), control rats without treatment; Ctrl+GW (*n* = 6–7), GW0742-treated control rats; TLE+Veh (*n* = 6–7), TLE rats; TLE+GW (*n* = 5–7), GW0742-treated TLE rats. Data are presented as mean and standard error of the mean. Each point represents the value of a single animal. Asterisks indicate significant differences between groups according to Sidak’s post hoc test: * *p* < 0.05; ** *p* < 0.01; *** *p* < 0.001. ANOVA contrast method: # *p* = 0.05.

The NOD-like receptor protein 3 (NLRP3), a basic inflammasome protein, has been identified as another protein associated with the triggering of pro-inflammatory signaling pathways [43]. The primary sources of NLRP3 in the brain are microglial cells [44]. The expression of the *Nlrp3* gene was observed to increase by more than twofold in both the dorsal hippocampus and the cortex (TLE factor: F_(1, 24)_ = 44, *p* < 0.001 and F_(1, 21)_ = 88, *p* < 0.001) (Figure 6a,b). Furthermore, in the cortex, GW0742 treatment resulted in a reduction in *Nlrp3* gene expression (treatment factor: F_(1, 21)_ = 5.1, *p* = 0.03), and the application of ANOVA contrast method also revealed differences between the TLE+Veh and TLE+GW groups (*p* = 0.05).

Subsequently, the gene expression of two microglial proteins was examined, namely the inducible NO synthase (*Nos2* gene, Figure 6c,d) and the arginase enzyme (*Arg1* gene, Figure 6e,f). It is established that in macrophages and microglia, these proteins compete for the substrate L-arginine, which can activate the intracellular cascades associated with the production of damaging (*Nos2*) or protective (*Arg1*) factors, respectively [45]. Consequently, an additional analysis was conducted on the *Nos2/Arg1* ratio as an indicator of the equilibrium between damaging and neuroprotective mechanisms (Figure 6g,h).

No significant alterations in the *Nos2* gene expression were observed in the studied brain structures. In the dorsal hippocampus, *Arg1* gene expression was significantly decreased (TLE factor: F_(1, 21)_ = 12.0, *p* = 0.002) and the *Nos2*/*Arg1* ratio increased (TLE factor: F_(1, 24)_ = 5.7; *p* = 0.02). In both cases, post hoc comparisons reveal significant changes only in untreated rats.

#### 2.3.4. Analysis of the Expression of Astroglial Proteins

**Figure 7:**
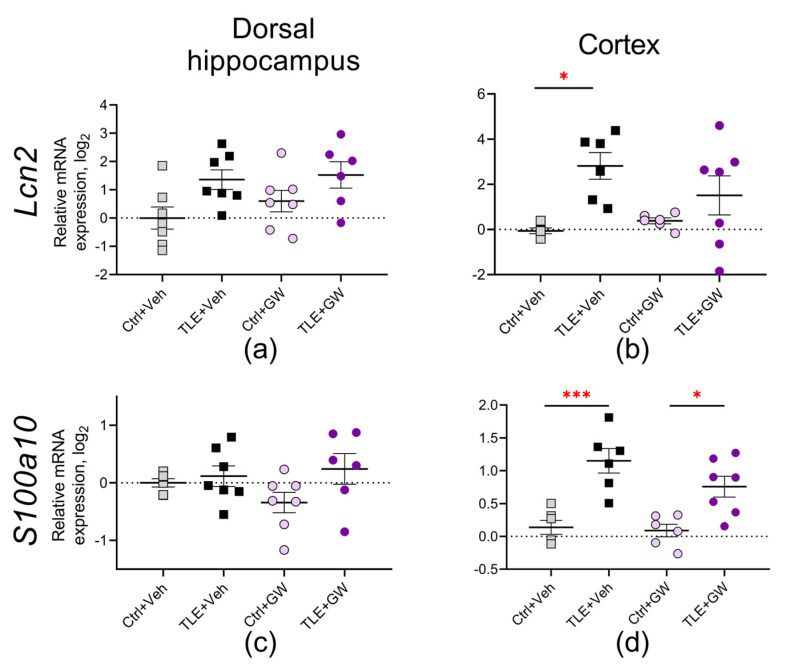
Relative gene expression of astroglial proteins *Lcn2* (**a**,**b**) and *S100a10* (**c**,**d**) in the dorsal hippocampus and cortex of experimental and control rats. Ctrl+Veh (*n* = 7), control rats without treatment; Ctrl+GW (*n* = 6–7), GW0742-treated control rats; TLE+Veh (*n* = 6–7), TLE rats; TLE+GW (*n* = 6–7), GW0742-treated TLE rats. Data are presented as mean and standard error of the mean. Each point represents the value of a single animal. Asterisks indicate significant differences between groups according to Sidak’s test: * *p* < 0.05; *** *p* < 0.001.

Next, we analyzed the expression of the following two astrocytic protein genes: lipocalin (*Lcn2* gene, Figure 7a,b), which plays an important role in the induction of neuronal death in inflammatory and pathological conditions of the CNS [46,47] and a marker of the protective phenotype of astroglia calcium-binding protein S100A10 (*S100a10* gene, Figure 7c,d). *Lcn2* gene expression was increased in the dorsal hippocampus (TLE factor: F_(1, 23)_ = 8.417; *p* < 0.01) and in the cortex (TLE factor: F_(1, 20)_= 11.07; *p* < 0.01). Nevertheless, post hoc comparisons indicate a statistically significant increase exclusively in the cortex of the untreated TLE rats. The expression of the *S100a10* gene was increased in the cortex of both the treated and untreated TLE animals (TLE factor: F_(1, 21)_ = 34; *p* < 0.001).

**Figure 8:**
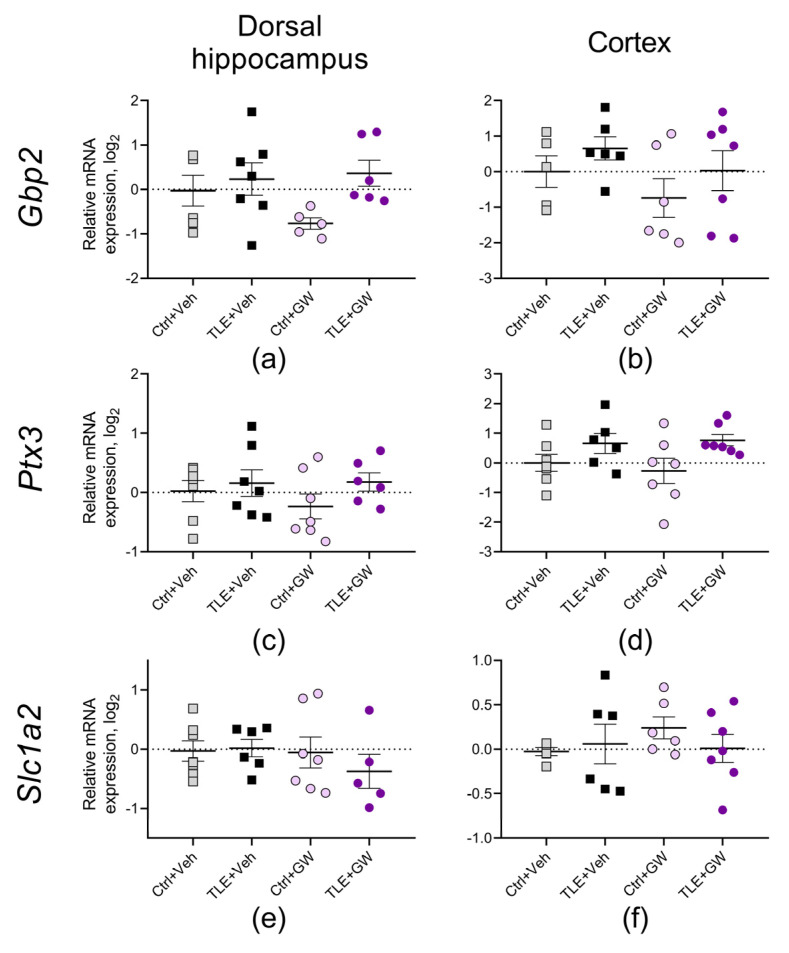
Relative gene expression of astroglial proteins (**a**–**f**) in the dorsal hippocampus and cortex of experimental and control rats. Ctrl+Veh (*n* = 7), control rats without treatment; Ctrl+GW (*n* = 5–7), GW0742-treated control rats; TLE+Veh (*n* = 6–7), TLE rats; TLE+GW (*n* = 6–7), GW0742-treated TLE rats. Data are presented as mean and standard error of the mean. Each point represents the value of a single animal.

In addition, the expression of three astroglial protein genes that may be associated with epileptogenesis were examined, namely guanylate binding protein 2 (*Gbp2* gene) [48,49] pentraxin-3 (*Ptx3*) [49,50], and the glutamate transporter EAAT2 (*Slc1a2*) [51,52]. No significant differences were observed between the groups regarding these genes (Figure 8).

## 3. Discussion

In this study, we investigated the effects of the PPARβ/δ agonist GW0742 on the progression of epileptogenesis in a rat lithium–pilocarpine model of TLE.

TLE resulted in impaired social and motor activity and affected the activation of astrocytes and microglial cells, as determined by analyzing the expression levels of a number of glial genes. Treatment with GW0742 attenuated some of these impairments but did not completely block them. Concurrently, the administration of GW0742 to control animals did not result in any observable alterations in their behavior or gene expression.

PPARβ/δ agonists, with GW0742 as one such agonist, have been demonstrated to possess pronounced neuroprotective and anti-inflammatory properties [25,39]. Given the pivotal role of neuroinflammation in the mechanisms of epileptogenesis [53], PPARβ/δ agonists may emerge as a valuable therapeutic tool for the treatment and prevention of epilepsy. Moreover, PPARβ/δ agonists have been demonstrated to exert a significant influence on oxidative stress [54,55] and lipid and carbohydrate metabolism [56]. These effects may potentially contribute to the inhibition of epileptogenesis [57,58,59]. For example, our recent findings demonstrated that cardarin, a PPARβ/δ agonist, effectively mitigates the behavioral disturbances that emerge during the chronic phase of the TLE model [60].

The effects of the PPARβ/δ agonist GW0742 were examined in the latent phase of the TLE model. One week following pilocarpine administration, the rats exhibited hyperactivity in the open field test and a significant reduction in communicative behavior in the social test. These behavioral changes are characteristic of the model used and have been consistently reproduced in our studies [9,61,62] and in other laboratories [63,64,65]. The behavioral abnormalities observed in the lithium–pilocarpine model may be specifically related to neuroinflammation, as evidenced by the attenuation of these abnormalities by anti-inflammatory therapy [9]. The present study showed that GW0742 did not completely block the behavioral deficits; however, in TLE rats treated with GW0742, motor activity in the open field test was not significantly increased.

The effects of GW0742 and the other PPARβ/δ agonists are thought to be related to their effects on astroglial and microglial cells [66,67,68]. The present study demonstrates that the utilized model of TLE activates the glial cells. During the latent phase of the lithium–pilocarpine model, we observed that *Gfap* and *Aif1* are upregulated at both the mRNA and protein levels in the dorsal hippocampus and cortex. The upregulation of these genes has been identified as an indicator of astrogliosis and microgliosis and has been demonstrated in numerous models of seizures and epilepsy [69,70,71,72,73].

The colony-stimulating factor 1 receptor (CSF1R)-related pathway has been identified as a key regulator of microglial activation [70], while astroglial activation has been linked to an increase in JAK2 tyrosine kinase and STAT3 protein production [69]. The latter pathway is subject to regulation by PPARs [74]. This study is the first to demonstrate that GW0742 treatment can attenuate the increased expression of *Gfap* and *Aif1* genes at the mRNA and protein levels in a TLE model. Notably, similar results have been reported in the treatment of epilepsy with a ketogenic diet [75], the effects of which are presumably mediated by PPARs [76].

The impact of GW0742 on *Gfap* and *Aif1* gene expression is of functional significance, as microglia and astrocyte activation contribute to epileptic processes [77]. One of the primary reasons for this is the close relationship between glial activation and the development of neuroinflammation [70], which plays a pivotal role in the pathogenic mechanisms of epileptogenesis [53]. The elevated gene expression of pro-inflammatory proteins, including *Il1b*, *Tnfa*, and *Nlrp3*, in the brains of rats with TLE, as observed in this study, is in accordance with the findings of previous studies conducted by our research group and other researchers [42,78,79]. It is noteworthy that, in the latent phase of the lithium–pilocarpine model, the expression of not only pro-inflammatory protein genes but also *Il1rn,* the gene encoding the anti-inflammatory cytokine, was increased. Furthermore, the production of *Il1rn* mRNA was more prominent than that of *Il1b*, as indicated by a reduction in the *Il1b/Il1rn* ratio. GW0742 diminished the elevated *Il1rn* gene expression in the cortex. It is plausible that the augmentation of *Il1rn* expression is contingent upon elevated glial cell activation, and this was mitigated following GW0742 administration. In planning this study, we hypothesized that GW0742 might attenuate the gene expression of pro-inflammatory cytokines. However, no convincing evidence was obtained in this study. In models of epilepsy, the effect of GW0742 on pro-inflammatory cytokine gene expression has not been previously studied; however, in other neuropathological models, administration of GW0742 has been shown to inhibit the enhanced expression of *Il1b* and *Tnfa* genes in brain cells induced by intracerebral hemorrhage and whole brain irradiation [39,80].

Furthermore, we investigated the gene expression of several proteins that are traditionally regarded as markers of polar (pro-inflammatory or protective) functional states of microglia and astrocytes. The classification of microglia and astrocytes into the M1/M2 and A1/A2 phenotypes has been the subject of criticism, as these states are rarely observed in pure form [15]. Nevertheless, even outside this classification framework, the genes under consideration are of interest for research purposes, as they play a role in regulating the damaging or neuroprotective processes during epileptogenesis.

Our findings demonstrate that a TLE-induced increase in the expression of the lipocalin-2 gene (*Lcn2*, a marker of A1 astrocytes) occurs in the rat brain. Lipocalin-2 is an iron transport protein that contributes to oxidative stress and inflammation [81]. Elevated levels of lipocalin-2 are considered a biomarker of brain injury [82,83]. Previous studies have demonstrated a significant elevation in lipocalin-2 levels in the hippocampus of wild-type mice subjected to a kainate-induced seizure model [81]. Conversely, lipocalin-2 deficiency in knockout mice has been shown to mitigate kainate-induced iron overload and oxidative stress in the hippocampal cells [81]. In our experiment, GW0742 did not significantly affect *Lcn2* gene expression.

We also analyzed the gene expression of the A2 phenotype astrocyte marker gene *S100a10*; it was increased in both the treated and untreated rats in the hippocampus and cortex during the latent phase of epileptogenesis. S100A10 is a polyfunctional protein; in complex with annexin A2, it is involved in the organization of lipid microdomains on the cell membrane, binding of actin filaments and cytoskeleton scaffolds, in membrane transport and fibrinolysis [84,85,86], and in the regulation of the activity of some ion channels [87]. The neuroprotective role of S100A10 in epilepsy may be related, in particular, to its effect on the serotoninergic system [87], which plays a protective role in epileptogenesis [88]. Previously, an enhanced expression of the *S100a10* gene in the hippocampus was shown in rats in a kainate model of TLE [89]. It is likely that the increased expression of the *S100a10* gene is associated with the triggering of neuroprotective mechanisms at the initial stages of epileptogenesis.

We also found decreased *Arg1* gene expression (a marker of M2 microglia) and an increased ratio of the M1 marker *Nos2* to *Arg1* in the hippocampus of the untreated TLE rats. It is known that macrophages and related microglial cells are characterized by alternative pathways of arginine metabolism via inducible NO synthase or arginase with different functional consequences [45]. The first pathway leads to NO synthesis, cell death due to oxidative stress, impaired energy metabolism, DNA damage, and other negative consequences [90]. The second pathway is associated with the increased synthesis of ornithine decarboxylase and consequent attenuation of inflammation [91]. Our study shows that no decrease in *Arg1* gene expression was observed in the treated TLE rats, which may indicate a neuroprotective effect of GW0742.

The expression of glutamate transporter genes EAAT2 (gene is *Slc1a2*), astroglia marker A1 guanylate-binding protein 2 (*Gbp2*), and astroglia marker A2 pentraxin-3 (*Ptx3*) was not altered in rat brain during the latent phase of the TLE model in this experiment. For the *Slc1a2* and *Ptx3* genes, similar results were obtained in our previous studies [9,92]. Increased *Gbp2* gene expression in the rat cortex was previously detected in the chronic phase of the lithium–pilocarpine model [92]. GW0742 did not affect the expression of the listed genes.

It is important to note the limitations of the study conducted. To avoid contradictions in the obtained data, it was decided to conduct the study on males only, since it is known that changes in this model depend on the sex of the animals [93,94]. Future research is planned to be conducted on females as well. Another limitation of this manuscript is that only one dose of GW0742 was used in the study. In addition, the study only evaluated the effects of the drug in the latent phase of the model. Similar studies have not been conducted in the chronic phase of the lithium–pilocarpine model. Further investigations into the chronic period of the model and dose–response analysis are planned for the future.

## 4. Materials and Methods

### 4.1. Experimental Design

**Figure 9:**
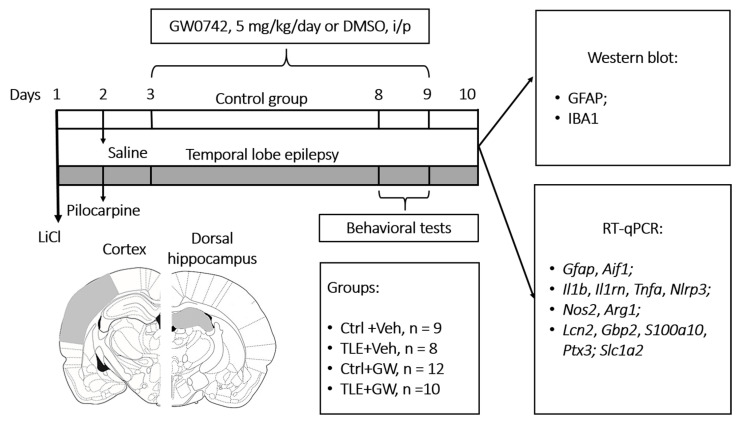
Design of the experiment and scheme of brain regions dissection for analysis. Ctrl+Veh, control rats without treatment; Ctrl+GW, GW0742-treated control rats; TLE+Veh, TLE rats; TLE+GW, GW0742-treated TLE rats.

The experimental design is illustrated in Figure 9. The studies were conducted on male Wistar rats aged 7–8 weeks. The lithium–pilocarpine model was utilized for the purpose of modeling TLE. This experimental model accurately reproduces the pathophysiological, histopathological, and biochemical processes associated with the development of human TLE [83]. The study was conducted during the initial period of epileptogenesis, which is the latent period of the model and coincides with the absence of spontaneous recurrent seizures.

Rats were intraperitoneally (i.p.) administered 127 mg/kg of LiCl (Sigma-Aldrich, St. Louis, MO, USA). On the subsequent day, pilocarpine (Sigma-Aldrich) was administered to induce convulsions. One hour prior to administration, an injection of (-) scopolamine methyl bromide (1 mg/kg, i.p.; Sigma-Aldrich) was administered to prevent the peripheral effects of pilocarpine. The dosage of pilocarpine was selected on an individual basis, based on the observed reaction of the rats to its administration. Pilocarpine was initially administered at a dose of 10 mg/kg (i.p.), with subsequent doses administered at 30 min intervals, until the development of stage 4 seizures, as defined by the Racine scale (see Appendix A Table A1) [95]. The requisite dose of pilocarpine for the induction of such convulsions was observed to be within the range of 20 to 40 mg/kg. It has been demonstrated that fractional administration of pilocarpine enhances animal survival, with the formation of temporal lobe epilepsy occurring in all rats that had received the indicated dose range of pilocarpine [9]. Rats that did not develop seizures after the fourth injection (40 mg/kg) were excluded from the study, as this constituted no more than 5% of the total number of animals. Seizures were terminated 90 min after the onset of stage 4 by the administration of diazepam (10 mg/kg, i.p., Sigma-Aldrich). A total of four groups were established as follows: (1) untreated control (Ctrl+Veh; n = 9); (2) GW0742-treated control (Ctrl+GW0742; n = 8); (3) untreated TLE rats (TLE+Veh; n = 13; 12 survived); and (4) GW0742-treated TLE rats (TLE+GW0742; n = 15; 10 survived). In order to ensure an equal distribution of animals into the different groups, the dose of pilocarpine administered was taken into consideration. This ensured that the number of animals in the treated and untreated groups with TLE was balanced. However, the distribution of animals into groups was otherwise random.

GW0742 (5 mg/kg, i.p., Clearsynth, Mumbai, India) was administered in a single daily dose (in a volume of 0.2 mL per 100 g) for a period of one week following the induction of status epilepticus by pilocarpine. The initial injection was administered 24 h following pilocarpine administration. Undiluted dimethyl sulfoxide (DMSO) was utilized as the solvent, given that GW0742 is essentially insoluble in water. The control animals were administered with DMSO. The dosage of the drug was selected based on the effectiveness of the doses in the treatment of other types of neuropathology, the pathogenesis of which is associated with neuroinflammation—brain injury, ischemia, neurodegenerative diseases [25].

The weight and survival of the animals were assessed during the whole period of drug administration. To increase the survival rate of TLE rats, in the first days after pilocarpine-induced epileptic status, they were fed with water from a pipette and wet food (porridge, fruit).

### 4.2. Behavioral Tests

Behavioral testing was conducted on the 7th and 8th days following pilocarpine administration, utilizing the open field and social tests (Figure 9).

The open field test was conducted in a circular arena with a diameter of 1 m and walls that were 30 cm in height. The illumination of the experimental setup was 8 lux. The rat was positioned in the center of the field and its behavior was recorded for a period of five minutes via video. Subsequently, the behavior was analyzed using the Tracking and Field V4.0 software (Institute of Experimental Medicine, St. Petersburg, Russia). Locomotor activity (quantified in terms of distance traveled and time spent in motion) and an indicator of psycho-emotional tension (measured in terms of the duration of grooming behavior) were evaluated.

The social interaction test was conducted in transparent Plexiglas cages measuring 60 × 30 × 40 cm. The male rat was placed in the cage setup one day prior to the test to allow for adaptation and space exploration. Another adult male rat was then placed into the experimental cage for a five-minute observation period. During this time, the cage host’s communicative (sniffing and grooming of the partner) and aggressive behaviors were evaluated.

### 4.3. Real-Time RT-PCR

Following the completion of behavioral testing, the animals were euthanized, and their brains were removed and stored at −80 °C until the subsequent biochemical studies could be conducted. The cortex (includes areas of temporal and parietal cortex) and dorsal hippocampus (Figure 9) were isolated from the brain using a cryostat microtome (OTF5000, Bright Instrument, Luton, UK). The required brain regions were identified using a rat brain atlas [96]. The cortex and dorsal hippocampus were selected for study due to their critical involvement in the pathogenesis of epilepsy [97]. Half of each rat’s brain was used for RT-qPCR and the other half for Western blotting. The method employed for the analysis of each brain hemisphere was selected at random.

Total RNA was isolated using ExtractRNA reagent (Evrogen, Moscow, Russia) according to the manufacturer’s recommendations. RNA concentration and purity were measured on a NanoDrop™ Lite spectrophotometer (ThermoFisher Scientific, Waltham, MA, USA), by absorbance at the 260 nm and 260/280 nm ratio, respectively.

cDNA synthesis was performed using 1 μg of total RNA, oligo-dT (0.5 μg per 1 μg RNA) and 9-mer random primers (0.25 μg per 1 μg RNA, DNA Synthesis LLC, Moscow, Russia) and 100 units of M-MLV reverse transcriptase (Evrogen). The reaction was performed in a total volume of 20 μL, and the procedure was carried out according to the recommendations of the manufacturers. Before further PCR, the obtained cDNA was diluted 10-fold.

Next, we analyzed gene expression of the following proteins that may influence the processes of epileptogenesis: markers of astroglial (*Gfap*) and microglial (*Aif1*) cell activation; pro-inflammatory and anti-inflammatory proteins (*Nlrp3, Il1b*, *Tnfa, Il1rn*); markers of pro-inflammatory and anti-inflammatory phenotypes of microglia (M1—*Nos2*; M2—*Arg1*); and markers of pro-inflammatory and neuroprotective phenotype of astroglia (A1—*Ptx3, Lcn2;* A2—*Gbp2*, *S100a10*), glutamate transporter *Slc1a2*. The expression of all the above genes was analyzed by real-time RT-PCR.

Real-time PCR (TaqMan technology) was performed on a C1000 Touch thermal cycler with a CFX384 Touch™ detector (Bio-Rad, Hercules, CA, USA). The reaction was performed in a total volume of 6 μL, with 0.8 μL of cDNA, 0.5 units of TaqM-polymerase (Alkor Bio, St. Petersburg, Russia), 3.5 mM MgCl_2_, and specific forward and reverse primers and probes (see Appendix A Table A2) synthesized by DNA-Synthesis LLC. Samples were analyzed in 4 repeats.

The relative expression of the genes of interest was calculated using the method of 2^−∆∆Ct^ [98]. Normalization was performed using the geometric mean of the expression of the three most stable reference genes selected from nine reference genes (*Gapdh, Actb, Rpl13a, B2m, Pgk1, Ppia, Hprt1, Ywhaz, Sdha*). Reference genes were selected based on the ranking obtained using the online tool RefFinder^®^ (http://blooge.cn/RefFinder/ accessed on 10 May 2024) according to the procedure described previously [99]. In the conducted study, the following reference genes were used for data normalization: *Actb*, *Rpl13a,* and *Pgk1* for dorsal hippocampus; *Sdha, Gapdh,* and *Ppia* for cortex.

### 4.4. Western Blot Analysis

The isolated brain structures were homogenized on ice in an optimized lysis buffer proposed [100], containing 100 mM Tris-HCl pH 8.0, 140 mM NaCl, 20 mM EDTA, 5% dodecyl sodium sulfate, 1X protease inhibitor cocktail (Pierce Protease Inhibitor Tablets, ThermoFisher Scientific), 1 mM sodium orthovanadate, and 20 mM sodium fluoride. The homogenate was incubated for one hour at room temperature with constant stirring. The samples were then centrifuged (15 min, 14,000× *g*, 20 °C), and the supernatant was used for protein concentration quantification and Western blotting. Protein concentration was determined by the Lowry method as modified by Hartree [101]. The supernatant was diluted 1:1 with 2× application buffer (125 mM Tris-HCl pH 6.8, 40% glycerol, 4% sodium dodecyl sulfate, 5% β-mercaptoethanol, 0.02% bromphenol blue) and incubated for 15 min at 70 °C and then stored at −20 °C until electrophoresis.

Prior to electrophoresis, the protein concentration was equalized by diluting the samples with 1× plating buffer.

Electrophoretic separation was performed under reducing and denaturing conditions [102] in a 13.5% polyacrylamide gel together with a molecular weight standard (Thermo Scientific PageRuler Prestained Protein Ladder 10–170 kDA; ThermoFisher Scientific) at an amperage of 125 V. A total of 12 μg of protein was taken for plating because this concentration allowed our samples to fall within the linear region of the densitometric analysis for all the antibodies used under our conditions. Together with the analyzed samples, a calibrator sample obtained by mixing several samples from animals from different groups was applied to each gel.

Proteins were transferred onto a nitrocellulose membrane (0.2 μm pore diameter) by semi-dry transfer with the Invitrogen Power Blotter 1-Step Transfer Buffer (ThermoFisher Scientific), according to the manufacturer’s instructions, for 13 min at a current of 2.5 A. After transfer, the membrane was stained with a 0.1% solution of Ponseau S dye in 5% acetic acid and documented using a ChemiDoc MP gel imaging system (Bio-Rad). The membrane was then washed with phosphate-buffered saline (0.01 M phosphate buffer pH 7.4, 137 mM NaCl, 2.7 mM KCl) containing 0.1% Tween-20 (PBST). Blocking was performed using the SNAP I.D. 2.0 vacuum blot hybridization system (Merck Millipore, Burlington, MA, USA) in 0.5% skim milk powder solution (Sigma-Aldrich) diluted in PBST, according to the device manufacturer’s instructions. The membrane was then incubated overnight at +4 °C in a solution of primary antibodies in PBST containing 0.05% sodium azide against GFAP (ab7260, 1:10,000, rabbit polyclonal, Abcam, Cambridge, UK), Iba1 (1:1000, rabbit polyclonal, Novus Biologicals, Littleton, CO, USA), EAAT2 (ab205248, 1:1000, rabbit monoclonal, Abcam). The membrane was then washed with PBST from the primary antibodies, treated with a solution of secondary antibodies (antibodies against rabbit immunoglobulin G, cat. nom. 31460, 1:20,000, Pierce Goat anti-rabbit IgG-HRP, ThermoFisher Scientific) and washed from secondary antibodies using the SNAP I.D. 2.0 vacuum blot hybridization system (Merck Millipore) according to the manufacturer’s recommendations. Chemiluminescent signal was obtained using SuperSignal™ West Pico PLUS chemiluminescent substrate (ThermoFisher Scientific) and documented using the ChemiDoc MP system (Bio-Rad). Densitometric analysis was performed using the Image Lab 6.0.1 software (Bio-Rad); the optical specific signal was normalized to the calibrator sample by the Ponseau S (total protein) signal of the corresponding lane.

### 4.5. Statistical Analysis

The statistical analysis was conducted using the SPSS Statistics 23 software (IBM, Armonk, NY, USA) and the GraphPad Prism software version 8.4.3 (GraphPad Software, San Diego, CA, USA). Outliers were identified through the application of the quartile method. The log-rank test was employed for the analysis of survival curves in the rats. The Kolmogorov–Smirnov test was employed to ascertain the normality of the distributions. The assumption of homogeneity of variance was evaluated using the Levene test. A three-way mixed ANOVA (test days × TLE × treatment) was employed to analyze weight dynamics during drug administration. A mixed ANOVA (TLE × treatment × minute of test) was also used to analyze the dynamics of motor activity (distance travelled) in the open field test. For the remaining data, which exhibited a normal distribution, a two-way ANOVA (TLE × treatment) with Sidak’s post hoc multiple comparisons test was employed. The ANOVA contrast method was used to analyze the intergroup differences in more detail. Statistical significance was set at *p* ≤ 0.05. Graphs display the means and standard errors. The results of statistical processing are described in detail in the text for the variables for which statistically significant differences were identified.

## Data Availability

The data presented in this study are available upon request from the corresponding author.

## Appendix A

**Table A1:** Stages of the modified Racine scale [9,95].

Grade	Stage Description
1	Facial automatism
2	Head nodding
3	Forelimb myoclonus
4	Rearing
5	Rearing and falling
6	Wild running
7	Generalized clonic–tonic convulsions

**Table A2:** Primers and probes used in RT-qPCR.

Gene Symbol RefSeq Accession Number	Nucleotide Sequences (Forward, Reverse, TaqMan Probe)	Final Primers and Probe Concentration (nM)	Reference
*Actb* (actin beta) NM_031144	TGTCACCAACTGGGACGATA GGGGTGTTGAAGGTCTCAAA FAM-CGTGTGGCCCCTGAGGAGCAC-BHQ1	200 200	[103] (primers) [104] (probe)
*Gapdh* (glyceraldehyde-3-phosphate dehydrogenase) NM_017008	TGCACCACCAACTGCTTAG GGATGCAGGGATGATGTTC R6G-ATCACGCCACAGCTTTCCAGAGGG-BHQ2	200 100	[105]
*B2m* (beta-2-microglobulin) NM_012512	TGCCATTCAGAAAACTCCCC GAGGAAGTTGGGCTTCCCATT ROX-ATTCAAGTGTACTCTCGCCATCCACCG-BHQ1	200 100	[106]
*Rpl13a* (ribosomal protein L13a) NM_173340	GGATCCCTCCACCCTATGACA CTGGTACTTCCACCCGACCTC FAM-CTGCCCTCAAGGTTGTGCGGCT-BHQ1	200 100	[107] (primers) [104] (probe)
*Sdha* (succinate dehydrogenase complex flavoprotein subunit A) NM_130428	AGACGTTTGACAGGGGAATG TCATCAATCCGCACCTTGTA R6G-ACCTGGTGGAGACGCTGGAGCT-BHQ2	200 100	[108] (primers) [104] (probe)
*Ppia* (peptidylprolyl isomerase A) NM_017101	AGGATTCATGTGCCAGGGTG CTCAGTCTTGGCAGTGCAGA ROX-CACGCCATAATGGCACTGGTGGCA-BHQ1	200 100	[109]
*Hprt1* (hypoxanthine phosphoribosyltransferase 1) NM_012583	TCCTCAGACCGCTTTTCCCGC TCATCATCACTAATCACGACGCTGG FAM-CCGACCGGTTCTGTCATGTCGACCCT-BHQ1	200 100	[110] (primers) [104] (probe)
*Pgk1* (phosphoglycerate kinase 1) NM_053291	ATGCAAAGACTGGCCAAGCTAC AGCCACAGCCTCAGCATATTTC R6G-TGCTGGCTGGATGGGCTTGGA-BHQ2	200 100	[111] (primers) [104] (probe)
*Ywhaz* (tyrosine 3-monooxygenase/tryptophan 5-monooxygenase activation protein zet) NM_013011	GATGAAGCCATTGCTGAACTTG GTCTCCTTGGGTATCCGATGTC ROX-TGAAGAGTCGTACAAAGACAGCACGC-BHQ1	200 100	[111] (primers) [104] (probe)
*Il1b* (interleukin 1 beta) NM_031512	CACCTCTCAAGCAGAGCACAG GGGTTCCATGGTGAAGTCAAC FAM-TGTCCCGACCATTGCTGTTTCCTAG-BHQ1	400 200	[112]
*Tnfa* (tumor necrosis factor) NM_012675	CCAGGTTCTCTTCAAGGGACAA CTCCTGGTATGAAATGGCAAATC ROX-CCCGACTATGTGCTCCTCACCCACA-BHQ2	200 200	[112]
*Aif1* (allograft inflammatory factor 1) NM_017196.3	CAACACACTGCAGCCTCATC AAGCTTTTCCTCCCTGCAAA Cy5-CCCCACCTAAGGCCACCAGCGTCTGA-BHQ3	200 100	[42]
*Gfap* (glial fibrillary acidic protein) NM_017009.2	TGGCCACCAGTAACATGCAA CAGTTGGCGGCGATAGTCAT HEX-CGGTCCAAGTTTGCAGACCTCACAG-BHQ2	200 200	[113] (primers) [9] (probe)
*Slc1a2* (solute carrier family 1 member 2) NM_017215.2	CCAGTGCTGGAACTTTGCCT TAAAGGGCTGTACCATCCAT FAM-AGCGTGTGACCAGATTCGTCCTCCCA-BHQ1	200 150	[114] (primers) [9] (probe)
*Il1rn* (interleukin 1 receptor antagonist) NM_022194.2	GGGGACCTTACAGTCACCTAAT GGTTAGTATCCCAGATTCTGAAGG ROX-AGTCAGCTGGCCACCCTGCTGGGA-BHQ2	400 100	[42]
*Nlrp3* (NLR family pyrin domain containing 3) NM_001191642	CAGACCCTCATGTTGCCTGT AGACCTCGGCAGAAGCTAGA FAM-CCAGACTGGTGAACTGCTGCCTCA-BHQ1	200 100	[49]
*Lcn2* (lipocalin 2) NM_130741.1	AGCTACGATGTGCAAGTGGC CCCCTTGGTTCTTCCGTACA FAM-CGACACTGACTACGACCAGTTTGCCA-BHQ1	200 150	[49]
*Arg1* (arginase 1) NM_017134.3	AGCTGGGAATTGGCAAAGTG AACTCAGGTGAATGGGCCTT HEX-TGGAAGAGACCTTCAGCTACCTGC-BHQ2	300 100	[115] (primers) [49] (probe)
*S100a10* (S100 calcium binding protein A10) NM_031114.1	CATTTCACAGGTTTGCAGGGG GCACTGGTCCAGGTCTTTCA Cy5-AGGACCCTCTGGCTGTGGACA-BHQ3	200 250	[49]
*Ptx3* (pentraxin 3) NM_001109536.2	AAACTTCGCCTCTCCAGCAA CATGGTGTGGGGTCCTCG HEX-TGCTCTCTGGTCTGCAGTGTTGGC-BHQ2	400 200	[49]
*Gbp2* (guanylate binding protein 2) NM_133624.2	AGTCAATGGGCCACGTCTAA AGTGGGTGATGGCCTTTTGT HEX-AGCAGTGGGTCTCTCCCCTGCA-BHQ2	200 100	[49]
*Nos2* (nitric oxide synthase 2) NM_012611.3	CAGAAGCAGAATGTGACCATCAT CGGAGGGACCAGCCAAATC ROX-CCACCACACAGCCTCAGAGTCCTT-BHQ2	200 200	[116]

## References

[1] Banerjee P.N., Filippi D., Hauser W.A. (2009). The descriptive epidemiology of epilepsy—A review. Epilepsy Res..

[2] Fisher R.S., Acevedo C., Arzimanoglou A., Bogacz A., Helen Cross J., Elger C.E., Engel Jr J., Forsgren L., French J.A., Glynn M. (2014). A practical clinical definition of epilepsy. Epilepsia.

[3] Hakami T. (2021). Neuropharmacology of Antiseizure Drugs. Neuropsychopharmacol. Rep..

[4] Löscher W., Klitgaard H., Twyman R.E., Schmidt D. (2013). New avenues for anti-epileptic drug discovery and development. Nat. Rev. Drug Discov..

[5] Sanz P., Rubio T., Garcia-Gimeno M.A. (2024). Neuroinflammation and Epilepsy: From Pathophysiology to Therapies Based on Repurposing Drugs. Int. J. Mol. Sci..

[6] Devinsky O., Vezzani A., Najjar S., De Lanerolle N.C., Rogawski M.A. (2013). Glia and epilepsy: Excitability and inflammation. Trends Neurosci..

[7] Riazi K., Galic M.A., Kuzmiski J.B., Ho W., Sharkey K.A., Pittman Q.J. (2008). Microglial activation and TNFα production mediate altered CNS excitability following peripheral inflammation. Proc. Natl. Acad. Sci. USA.

[8] Rodgers K.M., Hutchinson M.R., Northcutt A., Maier S.F., Watkins L.R., Barth D.S. (2009). The cortical innate immune response increases local neuronal excitability leading to seizures. Brain.

[9] Dyomina A.V., Zubareva O.E., Smolensky I.V., Vasilev D.S., Zakharova M.V., Kovalenko A.A., Schwarz A.P., Ischenko A.M., Zaitsev A.V. (2020). Anakinra Reduces Epileptogenesis, Provides Neuroprotection, and Attenuates Behavioral Impairments in Rats in the Lithium–Pilocarpine Model of Epilepsy. Pharmaceuticals.

[10] Lee S.-H., Han S.-H., Lee K.-W. (2000). Kainic acid-induced seizures cause neuronal death in infant rats pretreated with lipopolysaccharide. Neuroreport.

[11] Suleymanova E.M. (2021). Behavioral comorbidities of epilepsy and neuroinflammation: Evidence from experimental and clinical studies. Epilepsy Behav..

[12] Li D., Wang Y., Guo Y., Wang W. (2024). Bioinformatics analysis reveals multiple functional changes in astrocytes in temporal lobe epilepsy. Brain Res..

[13] Guo S., Wang H., Yin Y. (2022). Microglia Polarization From M1 to M2 in Neurodegenerative Diseases. Front. Aging Neurosci..

[14] Fan Y.-Y., Huo J. (2021). A1/A2 astrocytes in central nervous system injuries and diseases: Angels or devils?. Neurochem. Int..

[15] Paolicelli R.C., Sierra A., Stevens B., Tremblay M.-E., Aguzzi A., Ajami B., Amit I., Audinat E., Bechmann I., Bennett M. (2022). Microglia states and nomenclature: A field at its crossroads. Neuron.

[16] Li P., Ji X., Shan M., Wang Y., Dai X., Yin M., Liu Y., Guan L., Ye L., Cheng H. (2023). Melatonin regulates microglial polarization to M2 cell via RhoA/ROCK signaling pathway in epilepsy. Immun. Inflamm. Dis..

[17] Zhao Q., Wu X., Yan S., Xie X., Fan Y., Zhang J., Peng C., You Z. (2016). The antidepressant-like effects of pioglitazone in a chronic mild stress mouse model are associated with PPARγ-mediated alteration of microglial activation phenotypes. J. Neuroinflamm..

[18] Yonutas H.M., Sullivan P.G. (2013). Targeting PPAR isoforms following CNS injury. Curr. Drug Targets.

[19] Grygiel-Górniak B. (2014). Peroxisome proliferator-activated receptors and their ligands: Nutritional and clinical implications—A review. Nutr. J..

[20] Li J., Guo C., Wu J. (2019). 15-Deoxy-∆-12,14-Prostaglandin J2 (15d-PGJ2), an Endogenous Ligand of PPAR-γ: Function and Mechanism. PPAR Res..

[21] Narala V.R., Adapala R.K., Suresh M.V., Brock T.G., Peters-Golden M., Reddy R.C. (2010). Leukotriene B4 is a physiologically relevant endogenous peroxisome proliferator-activated receptor-alpha agonist. J. Biol. Chem..

[22] Giordano Attianese G.M.P., Desvergne B. (2015). Integrative and systemic approaches for evaluating PPARβ/δ (PPARD) function. Nucl. Recept. Signal..

[23] Tufano M., Pinna G. (2020). Is There a Future for PPARs in the Treatment of Neuropsychiatric Disorders?. Molecules.

[24] Warden A., Truitt J., Merriman M., Ponomareva O., Jameson K., Ferguson L.B., Mayfield R.D., Harris R.A. (2016). Localization of PPAR isotypes in the adult mouse and human brain. Sci. Rep..

[25] Strosznajder A.K., Wójtowicz S., Jeżyna M.J., Sun G.Y., Strosznajder J.B. (2021). Recent Insights on the Role of PPAR-β/δ in Neuroinflammation and Neurodegeneration, and Its Potential Target for Therapy. Neuromol. Med..

[26] Genolet R., Wahli W., Michalik L. (2004). PPARs as drug targets to modulate inflammatory responses?. Curr. Drug Targets. Inflamm. Allergy.

[27] Senn L., Costa A.-M., Avallone R., Socała K., Wlaź P., Biagini G. (2023). Is the peroxisome proliferator-activated receptor gamma a putative target for epilepsy treatment? Current evidence and future perspectives. Pharmacol. Ther..

[28] Pérez-Segura I., Santiago-Balmaseda A., Rodríguez-Hernández L.D., Morales-Martínez A., Martínez-Becerril H.A., Martínez-Gómez P.A., Delgado-Minjares K.M., Salinas-Lara C., Martínez-Dávila I.A., Guerra-Crespo M. (2023). PPARs and Their Neuroprotective Effects in Parkinson’s Disease: A Novel Therapeutic Approach in α-Synucleinopathy?. Int. J. Mol. Sci..

[29] Yu X., Shao X.G., Sun H., Li Y.N., Yang J., Deng Y.C., Huang Y.G. (2008). Activation of cerebral peroxisome proliferator-activated receptors gamma exerts neuroprotection by inhibiting oxidative stress following pilocarpine-induced status epilepticus. Brain Res..

[30] Adabi Mohazab R., Javadi-Paydar M., Delfan B., Dehpour A.R. (2012). Possible involvement of PPAR-gamma receptor and nitric oxide pathway in the anticonvulsant effect of acute pioglitazone on pentylenetetrazole-induced seizures in mice. Epilepsy Res..

[31] Peng J., Wang K., Xiang W., Li Y., Hao Y., Guan Y. (2019). Rosiglitazone polarizes microglia and protects against pilocarpine-induced status epilepticus. CNS Neurosci. Ther..

[32] Peng Y., Chen L., Qu Y., Wang D., Zhu Y., Zhu Y. (2021). Rosiglitazone Prevents Autophagy by Regulating Nrf2-Antioxidant Response Element in a Rat Model of Lithium-pilocarpine-induced Status Epilepticus. Neuroscience.

[33] Puligheddu M., Pillolla G., Melis M., Lecca S., Marrosu F., De Montis M.G., Scheggi S., Carta G., Murru E., Aroni S. (2013). PPAR-Alpha Agonists as Novel Antiepileptic Drugs: Preclinical Findings. PLoS ONE.

[34] Sznaidman M.L., Haffner C.D., Maloney P.R., Fivush A., Chao E., Goreham D., Sierra M.L., LeGrumelec C., Xu H.E., Montana V.G. (2003). Novel selective small molecule agonists for peroxisome proliferator-activated receptor delta (PPARdelta)—Synthesis and biological activity. Bioorg. Med. Chem. Lett..

[35] Nandhikonda P., Yasgar A., Baranowski A.M., Sidhu P.S., McCallum M.M., Pawlak A.J., Teske K., Feleke B., Yuan N.Y., Kevin C. (2013). Peroxisome proliferation-activated receptor δ agonist GW0742 interacts weakly with multiple nuclear receptors, including the vitamin D receptor. Biochemistry.

[36] Niu H.-S., Ku P.-M., Niu C.-S., Cheng J.-T., Lee K.-S. (2015). Development of PPAR-agonist GW0742 as antidiabetic drug: Study in animals. Drug Des. Devel. Ther..

[37] Di Paola R., Esposito E., Mazzon E., Paterniti I., Galuppo M., Cuzzocrea S. (2010). GW0742, a selective PPAR-beta/delta agonist, contributes to the resolution of inflammation after gut ischemia/reperfusion injury. J. Leukoc. Biol..

[38] Haskova Z., Hoang B., Luo G., Morgan L.A., Billin A.N., Barone F.C., Shearer B.G., Barton M.E., Kilgore K.S. (2008). Modulation of LPS-induced pulmonary neutrophil infiltration and cytokine production by the selective PPARbeta/delta ligand GW0742. Inflamm. Res..

[39] Tang X., Yan K., Wang Y., Wang Y., Chen H., Xu J., Lu Y., Wang X., Liang J., Zhang X. (2020). Activation of PPAR-β/δ Attenuates Brain Injury by Suppressing Inflammation and Apoptosis in a Collagenase-Induced Intracerebral Hemorrhage Mouse Model. Neurochem. Res..

[40] Das N.R., Gangwal R.P., Damre M.V., Sangamwar A.T., Sharma S.S. (2014). A PPAR-β/δ agonist is neuroprotective and decreases cognitive impairment in a rodent model of Parkinson’s disease. Curr. Neurovasc. Res..

[41] An Y.-Q., Zhang C.T., Du Y., Zhang M., Tang S.S., Hu M., Long Y., Sun H.B., Hong H. (2016). PPARδ agonist GW0742 ameliorates Aβ1-42-induced hippocampal neurotoxicity in mice. Metab. Brain Dis..

[42] Zubareva O.E., Dyomina A.V., Kovalenko A.A., Roginskaya A.I., Melik-Kasumov T.B., Korneeva M.A., Chuprina A.V., Zhabinskaya A.A., Kolyhan S.A., Zakharova M.V. (2023). Beneficial Effects of Probiotic Bifidobacterium longum in a Lithium-Pilocarpine Model of Temporal Lobe Epilepsy in Rats. Int. J. Mol. Sci..

[43] Kelley N., Jeltema D., Duan Y., He Y. (2019). The NLRP3 Inflammasome: An Overview of Mechanisms of Activation and Regulation. Int. J. Mol. Sci..

[44] Xiao L., Zheng H., Li J., Wang Q., Sun H. (2020). Neuroinflammation Mediated by NLRP3 Inflammasome After Intracerebral Hemorrhage and Potential Therapeutic Targets. Mol. Neurobiol..

[45] Rath M., Müller I., Kropf P., Closs E.I., Munder M. (2014). Metabolism via Arginase or Nitric Oxide Synthase: Two Competing Arginine Pathways in Macrophages. Front. Immunol..

[46] Kim J.H., Ko P.W., Lee H.W., Jeong J.Y., Lee M.G., Kim J.H., Lee W.H., Yu R., Oh W.J., Suk K. (2017). Astrocyte-derived lipocalin-2 mediates hippocampal damage and cognitive deficits in experimental models of vascular dementia. Glia.

[47] Bi F., Huang C., Tong J., Qiu G., Huang B., Wu Q., Li F., Xu Z., Bowser R., Xia X.-G. (2013). Reactive astrocytes secrete lcn2 to promote neuron death. Proc. Natl. Acad. Sci. USA.

[48] El Baassiri M.G., Rahal S.S., Fulton W.B., Sodhi C.P., Hackam D.J., Nasr I.W. (2023). Pharmacologic Toll-like receptor 4 inhibition skews toward a favorable A1/A2 astrocytic ratio improving neurocognitive outcomes following traumatic brain injury. J. Trauma Acute Care Surg..

[49] Zakharova M.V., Dyomina A.V., Kovalenko A.A., Zubareva O.E., Ischenko A.M., Zaitsev A.V. (2024). Anakinra Promotes M2 Microglia Activation during the Latent Phase of the Lithium-Pilocarpine Model of Temporal Lobe Epilepsy. J. Evol. Biochem. Physiol..

[50] Ding Z.-B., Song L.-J., Wang Q., Kumar G., Yan Y.-Q., Ma C.-G. (2021). Astrocytes: A double-edged sword in neurodegenerative diseases. Neural Regen. Res..

[51] Zaitsev A.V., Smolensky I.V., Jorratt P., Ovsepian S.V. (2020). Neurobiology, Functions, and Relevance of Excitatory Amino Acid Transporters (EAATs) to Treatment of Refractory Epilepsy. CNS Drugs.

[52] Zubareva O.E., Kovalenko A.A., Kalemenev S.V., Schwarz A.P., Karyakin V.B., Zaitsev A.V. (2018). Alterations in mRNA expression of glutamate receptor subunits and excitatory amino acid transporters following pilocarpine-induced seizures in rats. Neurosci. Lett..

[53] Soltani Khaboushan A., Yazdanpanah N., Rezaei N. (2022). Neuroinflammation and Proinflammatory Cytokines in Epileptogenesis. Mol. Neurobiol..

[54] Aleshin S., Reiser G. (2013). Role of the peroxisome proliferator-activated receptors (PPAR)-α, β/δ and γ triad in regulation of reactive oxygen species signaling in brain. Biol. Chem..

[55] Jimenez R., Toral M., Gómez-Guzmán M., Romero M., Sanchez M., Mahmoud A.M., Duarte J. (2018). The Role of Nrf2 Signaling in PPARβ/δ-Mediated Vascular Protection against Hyperglycemia-Induced Oxidative Stress. Oxid. Med. Cell. Longev..

[56] Magadum A., Engel F.B. (2018). PPARβ/δ: Linking Metabolism to Regeneration. Int. J. Mol. Sci..

[57] Chen Z.-P., Wang S., Zhao X., Fang W., Wang Z., Ye H., Wang M.-J., Ke L., Huang T., Lv P. (2023). Lipid-accumulated reactive astrocytes promote disease progression in epilepsy. Nat. Neurosci..

[58] Boison D., Steinhäuser C. (2018). Epilepsy and astrocyte energy metabolism. Glia.

[59] Geronzi U., Lotti F., Grosso S. (2018). Oxidative stress in epilepsy. Expert Rev. Neurother..

[60] Subkhankulov M.R., Sinyak D.S., Guk V.A., Postnikova T.Y., Roginskaya A.I., Zubareva O.E. (2024). Cardarin Effect on the Formation of Histopathological and Behavioral Abnormalities in the Lithium-Pilocarpine Model of Temporal Lobe Epilepsy in Rats. J. Evol. Biochem. Physiol..

[61] Dyomina A.V., Smolensky I.V., Zaitsev A. (2023). V Refinement of the Barnes and Morris water maze protocols improves characterization of spatial cognitive deficits in the lithium-pilocarpine rat model of epilepsy. Epilepsy Behav..

[62] Smolensky I.V., Zubareva O.E., Kalemenev S.V., Lavrentyeva V.V., Dyomina A.V., Karepanov A.A., Zaitsev A. (2019). V Impairments in cognitive functions and emotional and social behaviors in a rat lithium-pilocarpine model of temporal lobe epilepsy. Behav. Brain Res..

[63] Suleymanova E.M., Gulyaev M.V., Abbasova K.R. (2016). Structural alterations in the rat brain and behavioral impairment after status epilepticus: An MRI study. Neuroscience.

[64] Wulsin A.C., Franco-Villanueva A., Romancheck C., Morano R.L., Smith B.L., Packard B.A., Danzer S.C., Herman J.P. (2018). Functional disruption of stress modulatory circuits in a model of temporal lobe epilepsy. PLoS ONE.

[65] Rojas J.J., Deniz B.F., Miguel P.M., Diaz R., do Espírito-Santo Hermel É., Achaval M., Netto C.A., Pereira L.O. (2013). Effects of daily environmental enrichment on behavior and dendritic spine density in hippocampus following neonatal hypoxia-ischemia in the rat. Exp. Neurol..

[66] Pan H.-C., Yang C.-N., Lee W.-J., Sheehan J., Wu S.-M., Chen H.-S., Lin M.-H., Shen L.-W., Lee S.-H., Shen C.-C. (2024). Melatonin Enhanced Microglia M2 Polarization in Rat Model of Neuro-inflammation Via Regulating ER Stress/PPARδ/SIRT1 Signaling Axis. J. Neuroimmune Pharmacol..

[67] Chen L., Xue L., Zheng J., Tian X., Zhang Y., Tong Q. (2019). PPARß/δ agonist alleviates NLRP3 inflammasome-mediated neuroinflammation in the MPTP mouse model of Parkinson’s disease. Behav. Brain Res..

[68] Konttinen H., Gureviciene I., Oksanen M., Grubman A., Loppi S., Huuskonen M.T., Korhonen P., Lampinen R., Keuters M., Belaya I. (2019). PPARβ/δ-agonist GW0742 ameliorates dysfunction in fatty acid oxidation in PSEN1ΔE9 astrocytes. Glia.

[69] Xu Z., Xue T., Zhang Z., Wang X., Xu P., Zhang J., Lei X., Li Y., Xie Y., Wang L. (2011). Role of signal transducer and activator of transcription-3 in up-regulation of GFAP after epilepsy. Neurochem. Res..

[70] Feng L., Murugan M., Bosco D.B., Liu Y., Peng J., Worrell G.A., Wang H.L., Ta L.E., Richardson J.R., Shen Y. (2019). Microglial proliferation and monocyte infiltration contribute to microgliosis following status epilepticus. Glia.

[71] Kyriatzis G., Bernard A., Bôle A., Khrestchatisky M., Ferhat L. (2024). In the Rat Hippocampus, Pilocarpine-Induced Status Epilepticus Is Associated with Reactive Glia and Concomitant Increased Expression of CD31, PDGFRβ, and Collagen IV in Endothelial Cells and Pericytes of the Blood–Brain Barrier. Int. J. Mol. Sci..

[72] Dyomina A.V., Kovalenko A.A., Zakharova M.V., Postnikova T.Y., Griflyuk A.V., Smolensky I.V., Antonova I.V., Zaitsev A.V. (2022). MTEP, a Selective mGluR5 Antagonist, Had a Neuroprotective Effect but Did Not Prevent the Development of Spontaneous Recurrent Seizures and Behavioral Comorbidities in the Rat Lithium-Pilocarpine Model of Epilepsy. Int. J. Mol. Sci..

[73] Postnikova T.Y., Diespirov G.P., Amakhin D.V., Vylekzhanina E.N., Soboleva E.B., Zaitsev A.V. (2021). Impairments of Long-Term Synaptic Plasticity in the Hippocampus of Young Rats during the Latent Phase of the Lithium-Pilocarpine Model of Temporal Lobe Epilepsy. Int. J. Mol. Sci..

[74] Li J., Xiang X., Gong X., Shi Y., Yang J., Xu Z. (2017). Cilostazol protects mice against myocardium ischemic/reperfusion injury by activating a PPARγ/JAK2/STAT3 pathway. Biomed. Pharmacother..

[75] Lin D.T., Kao N.J., Cross T.W.L., Lee W.J., Lin S.H. (2022). Effects of ketogenic diet on cognitive functions of mice fed high-fat-high-cholesterol diet. J. Nutr. Biochem..

[76] Simeone T.A., Matthews S.A., Samson K.K., Simeone K.A. (2017). Regulation of brain PPARgamma2 contributes to ketogenic diet anti-seizure efficacy. Exp. Neurol..

[77] Robel S., Sontheimer H. (2015). Glia as drivers of abnormal neuronal activity. Nat. Neurosci..

[78] Qi R., Wang M., Zhong Q., Wang L., Yang X., Huang B., Yang Z., Zhang C., Geng X., Luo C. (2022). Chronic vagus nerve stimulation (VNS) altered IL-6, IL-1β, CXCL-1 and IL-13 levels in the hippocampus of rats with LiCl-pilocarpine-induced epilepsy. Brain Res..

[79] Pohlentz M.S., Müller P., Cases-Cunillera S., Opitz T., Surges R., Hamed M., Vatter H., Schoch S., Becker A.J., Pitsch J. (2022). Characterisation of NLRP3 pathway-related neuroinflammation in temporal lobe epilepsy. PLoS ONE.

[80] Schnegg C.I., Greene-Schloesser D., Kooshki M., Payne V.S., Hsu F.-C., Robbins M.E. (2013). The PPARδ agonist GW0742 inhibits neuroinflammation, but does not restore neurogenesis or prevent early delayed hippocampal-dependent cognitive impairment after whole-brain irradiation. Free Radic. Biol. Med..

[81] Shin H.J., Jeong E.A., Lee J.Y., An H.S., Jang H.M., Ahn Y.J., Lee J., Kim K.E., Roh G.S. (2021). Lipocalin-2 Deficiency Reduces Oxidative Stress and Neuroinflammation and Results in Attenuation of Kainic Acid-Induced Hippocampal Cell Death. Antioxidants.

[82] Liu R., Wang J., Chen Y., Collier J.M., Capuk O., Jin S., Sun M., Mondal S.K., Whiteside T.L., Stolz D.B. (2022). NOX activation in reactive astrocytes regulates astrocytic LCN2 expression and neurodegeneration. Cell Death Dis..

[83] Jha M.K., Suk K. (2013). Glia-based biomarkers and their functional role in the CNS. Expert Rev. Proteom..

[84] Hoellenriegel J., Meadows S.A., Sivina M., Wierda W.G., Kantarjian H., Keating M.J., Giese N., O’Brien S., Yu A., Miller L.L. (2011). The phosphoinositide 3′-kinase delta inhibitor, CAL-101, inhibits B-cell receptor signaling and chemokine networks in chronic lymphocytic leukemia. Blood.

[85] Babiychuk E.B., Draeger A. (2000). Annexins in cell membrane dynamics. Ca^2+^-regulated association of lipid microdomains. J. Cell Biol..

[86] Milosevic A., Liebmann T., Knudsen M., Schintu N., Svenningsson P., Greengard P. (2017). Cell- and region-specific expression of depression-related protein p11 (S100a10) in the brain. J. Comp. Neurol..

[87] Svenningsson P., Greengard P. (2007). p11 (S100A10)--an inducible adaptor protein that modulates neuronal functions. Curr. Opin. Pharmacol..

[88] Bagdy G., Kecskemeti V., Riba P., Jakus R. (2007). Serotonin and epilepsy. J. Neurochem..

[89] Dahal A., Govindarajan K., Kar S. (2023). Administration of Kainic Acid Differentially Alters Astrocyte Markers and Transiently Enhanced Phospho-tau Level in Adult Rat Hippocampus. Neuroscience.

[90] Murphy M.P. (1999). Nitric oxide and cell death. Biochim. Biophys. Acta.

[91] Hardbower D.M., Asim M., Luis P.B., Singh K., Barry D.P., Yang C., Steeves M.A., Cleveland J.L., Schneider C., Piazuelo M.B. (2017). Ornithine decarboxylase regulates M1 macrophage activation and mucosal inflammation via histone modifications. Proc. Natl. Acad. Sci. USA.

[92] Kharisova A.R., Roginskaya A.I., Zubareva O.E. (2024). Effect of Cardarine on Gene Expression of Proteins Involved in Epileptogenesis in Rat Hippocampus in the Lithium-Pilocarpine Model of Temporal Lobe Epilepsy. J. Evol. Biochem. Physiol..

[93] Matovu D., Cavalheiro E.A. (2022). Differences in Evolution of Epileptic Seizures and Topographical Distribution of Tissue Damage in Selected Limbic Structures Between Male and Female Rats Submitted to the Pilocarpine Model. Front. Neurol..

[94] Peternel S., Pilipović K., Zupan G. (2009). Seizure susceptibility and the brain regional sensitivity to oxidative stress in male and female rats in the lithium-pilocarpine model of temporal lobe epilepsy. Prog. Neuropsychopharmacol. Biol. Psychiatry.

[95] Racine R.J. (1975). Modification of seizure activity by electrical stimulation: Cortical areas. Electroencephalogr. Clin. Neurophysiol..

[96] Paxinos G., Watson C. (2006). The Rat Brain in Stereotaxic Coordinates: Hard Cover Edition.

[97] Chauhan P., Philip S.E., Chauhan G., Mehra S. (2022). The Anatomical Basis of Seizures. Epilepsy.

[98] Livak K.J., Schmittgen T.D. (2001). Analysis of Relative Gene Expression Data Using Real-Time Quantitative PCR and the 2−ΔΔCT Method. Methods.

[99] Schwarz A.P., Kovalenko A.A., Malygina D.A., Postnikova T.Y., Zubareva O.E., Zaitsev A.V. (2020). Reference gene validation in the brain regions of young rats after pentylenetetrazole-induced seizures. Biomedicines.

[100] Kopec A.M., Rivera P.D., Lacagnina M.J., Hanamsagar R., Bilbo S.D. (2017). Optimized solubilization of TRIzol-precipitated protein permits Western blotting analysis to maximize data available from brain tissue. J. Neurosci. Methods.

[101] Harrington C.R. (1990). Lowry protein assay containing sodium dodecyl sulfate in microtiter plates for protein determinations on fractions from brain tissue. Anal. Biochem..

[102] Laemmli U.K. (1970). Cleavage of structural proteins during the assembly of the head of bacteriophage T4. Nature.

[103] Bonefeld B.E., Elfving B., Wegener G. (2008). Reference genes for normalization: A study of rat brain tissue. Synapse.

[104] Schwarz A.P., Malygina D.A., Kovalenko A.A., Trofimov A.N., Zaitsev A.V. (2020). Multiplex qPCR assay for assessment of reference gene expression stability in rat tissues/samples. Mol. Cell. Probes.

[105] Lin W., Burks C.A., Hansen D.R., Kinnamon S.C., Gilbertson T.A. (2004). Taste Receptor Cells Express pH-Sensitive Leak K + Channels. J. Neurophysiol..

[106] Yamaguchi M., Yamauchi A., Nishimura M., Ueda N., Naito S. (2005). Soybean Oil Fat Emulsion Prevents Cytochrome P450 mRNA Down-Regulation Induced by Fat-Free Overdose Total Parenteral Nutrition in Infant Rats. Biol. Pharm. Bull..

[107] Swijsen A., Nelissen K., Janssen D., Rigo J.-M., Hoogland G. (2012). Validation of reference genes for quantitative real-time PCR studies in the dentate gyrus after experimental febrile seizures. BMC Res. Notes.

[108] Pohjanvirta R., Niittynen M., Lindén J., Boutros P.C., Moffat I.D., Okey A.B. (2006). Evaluation of various housekeeping genes for their applicability for normalization of mRNA expression in dioxin-treated rats. Chem. Biol. Interact..

[109] Malkin S.L., Amakhin D.V., Veniaminova E.A., Kim K.K., Zubareva O.E., Magazanik L.G., Zaitsev A. (2016). V Changes of AMPA receptor properties in the neocortex and hippocampus following pilocarpine-induced status epilepticus in rats. Neuroscience.

[110] Cook N.L., Vink R., Donkin J.J., van den Heuvel C. (2009). Validation of reference genes for normalization of real-time quantitative RT-PCR data in traumatic brain injury. J. Neurosci. Res..

[111] Langnaese K., John R., Schweizer H., Ebmeyer U., Keilhoff G. (2008). Selection of reference genes for quantitative real-time PCR in a rat asphyxial cardiac arrest model. BMC Mol. Biol..

[112] Rioja I., Bush K.A., Buckton J.B., Dickson M.C., Life P.F. (2004). Joint cytokine quantification in two rodent arthritis models: Kinetics of expression, correlation of mRNA and protein levels and response to prednisolone treatment. Clin. Exp. Immunol..

[113] Raghavendra V., Tanga F.Y., DeLeo J.A. (2004). Attenuation of Morphine Tolerance, Withdrawal-Induced Hyperalgesia, and Associated Spinal Inflammatory Immune Responses by Propentofylline in Rats. Neuropsychopharmacology.

[114] O’Donovan S.M., Hasselfeld K., Bauer D., Simmons M., Roussos P., Haroutunian V., Meador-Woodruff J.H., McCullumsmith R.E. (2015). Glutamate transporter splice variant expression in an enriched pyramidal cell population in schizophrenia. Transl. Psychiatry.

[115] Su J., Zhang Y., Cheng C., Zhu Y., Ye Y., Sun Y., Xiang S., Wang Y., Liu Z., Zhang X. (2021). Hydrogen regulates the M1/M2 polarization of alveolar macrophages in a rat model of chronic obstructive pulmonary disease. Exp. Lung Res..

[116] Sang N., Yun Y., Li H., Hou L., Han M., Li G. (2010). SO_2_ inhalation contributes to the development and progression of ischemic stroke in the brain. Toxicol. Sci..

